# Intussusception caused by small intestine metastasis of malignant pleural mesothelioma: a case report

**DOI:** 10.1093/jscr/rjab003

**Published:** 2021-02-22

**Authors:** Michinori Hamaoka, Masataka Nakagawa, Hideki Nakahara, Rie Yamamoto, Takashi Nishisaka, Toshiyuki Itamoto

**Affiliations:** Department of Gastroenterological Surgery, Hiroshima Prefectural Hospital, Hiroshima 734-8530, Japan; Department of Gastroenterological Surgery, Hiroshima Prefectural Hospital, Hiroshima 734-8530, Japan; Department of Gastroenterological Surgery, Hiroshima Prefectural Hospital, Hiroshima 734-8530, Japan; Department of Pathology, Hiroshima Prefectural Hospital, Hiroshima, Japan; Department of Pathology, Hiroshima Prefectural Hospital, Hiroshima, Japan; Department of Gastroenterological Surgery, Hiroshima Prefectural Hospital, Hiroshima 734-8530, Japan

## Abstract

Malignant pleural mesothelioma (MPM) is an aggressive form of malignant tumor that originates in the pleural mesothelioma and presents as a local disease in the affected hemithorax. Small intestine metastasis is a rare complication. Herein, the case of a patient with jejunal intussusception caused by small intestine metastasis of MPM has been reported. A 72-year-old man with MPM was admitted to our hospital for abdominal pain. Computed tomography revealed small intestine intussusception. An emergency surgery was performed, and the tumor and intussusception were located in the upper jejunum. Histopathological examination of the resected jejunum revealed that the tumor was a small intestinal metastasis of the MPM from the chest wall. This case showed that MPM may metastasize to the small intestine, and metastatic tumors may cause intussusception.

## INTRODUCTION

Malignant pleural mesothelioma (MPM) can invade both visceral and parietal pleura. It frequently extends to adjacent structures, such as the chest wall, mediastinum and diaphragm. Sites of lymph node spread and/or metastases include distant organs, such as the lungs, liver, kidneys, adrenal glands and brain. However, metastasis to the small intestine is rare [1]. Furthermore, intussusception caused by the metastatic MPM is a rare complication. Therefore, we present a rare case of a patient with jejunal intussusception caused by intestinal metastasis of MPM.

## CASE REPORT

**Figure 1:**
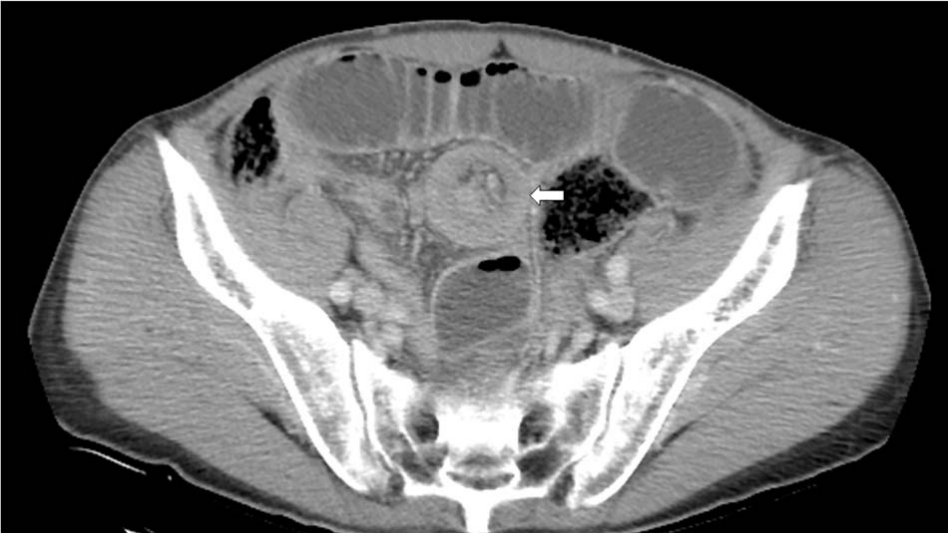
CT discloses a typical target sign over the small intestine (arrow).

A 72-year-old man was diagnosed with stage IV (cT4N0M0) epithelioid MPM with diffuse invasion of soft tissues of the right chest wall, according to the criteria of International Mesothelioma Interest Group, by computed tomography (CT), positron emission tomography (PET)-CT, and pleural biopsy 18 months ago. He was treated with pemetrexed and cisplatin for 12 months. Because of the appearance of lung metastasis, the patient was treated with nivolumab as second line therapy for 6 months. He complained of vague abdominal pain 2 months ago. He then underwent upper and lower gastrointestinal endoscopy, but no abnormalities were found. He was admitted to our hospital for aggravation of abdominal pain. His abdomen was flat and soft, and he complained of abdominal pain upon applying pressure on the entire abdomen. Hematological tests revealed a raised white blood cell count of 9400/μl and high C-reactive protein level of 10.09 mg/dl. CT scan revealed intussusception of the small intestine (Fig. 1).

An emergency surgery was performed. The tumor with the intussusception was located in the upper jejunum, 120 cm distal to the ligament of Treitz. After the intussusception was repositioned using the Hutchinson’s maneuver, the jejunum was resected. There was no thickening of the mesentery or disseminated nodules. The postoperative course was uneventful, and the patient was discharged on Day 9 post operation.

**Figure 2:**
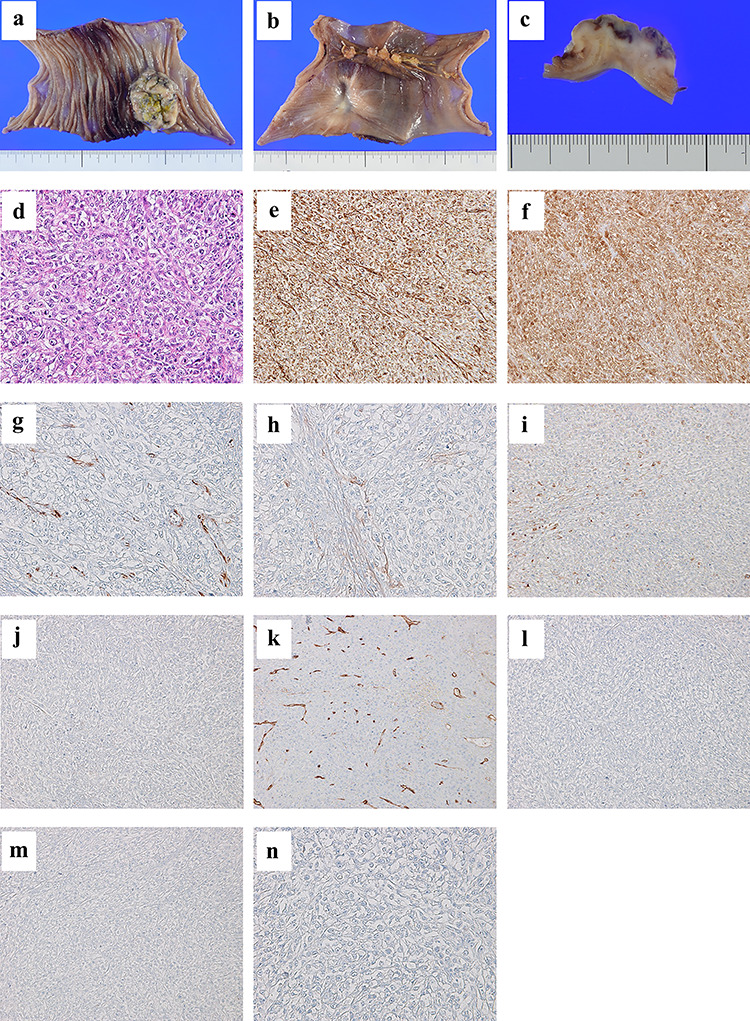
A tumor of size 35 mm is found from the mucosal to the subserosal layer of the jejunum (**a**, **b**, and **c**); the tumor is composed of sheets of epithelioid cells with high nuclear grade (×20 magnification hematoxylin and eosin staining) (**d**); immunohistochemical examination reveals tumor cells positive for vimentin (**e**), calretinin (**f**), WT-1 (**g**), D2-40 (**h**) and CK7 (**i**), and negative for CEA (**j**), CD34 (**k**), c-kit (**l**), CK5/6 (**m**) and CK20 (**n**).

Histopathological examination revealed mucosal and submucosal involvement. The tumor was composed of sheets of epithelioid cells with a high nuclear grade. On immunohistochemical examination, the tumor cells tested negative for CEA, CD34, c-kit, CK5/6 and C20, but they were positive for vimentin, calretinin, WT-1, D2-40 and CK7 (Fig. 2). The results were identical to those of the MPM from the chest wall. Thus, we concluded that the tumor was a metastasis of MPM.

## DISCUSSION

MPM is an aggressive form of malignant tumor that originates in the pleural mesothelioma. MPM typically invades surrounding organs and rarely metastasizes. However, upon reviewing autopsy cases, extrathoracic metastasis was found in 55% of the cases. The reported metastatic sites included almost all organs, such as the lymph nodes, lungs, adrenal glands, liver, kidneys and brain [1]. Metastases to the small intestine are infrequent. Upon reviewing published literature, seven cases of small intestine metastasis from MPM were found (Table 1) [2–8]. Diagnosing this condition before symptoms occur is difficult. In many cases, the diagnosis was made after the appearance of symptoms, such as anemia or perforation. Gocho *et al*. [4] have suggested the following reasons why detecting small intestine metastases is difficult. First, physicians have little knowledge of clinically rare metastases, such as small intestine metastases. Second, non-specific symptoms may be considered as general complaints or side effects of chemotherapy. Finally, follow-up CT scans have low sensitivity for small intestine tumors [4]. Similarly, the patient in our case complained of vague abdominal pain for ~2 months, and the small intestine metastasis could not be diagnosed before detecting the intussusception. Navaro *et al*. [7] have suggested that PET-CT as well as the combination of capsule endoscopy and double-balloon enteroscopy may aid in overcoming difficulties in detecting this type of metastasis. If patients with mesothelioma have unexplained abdominal pain, these tests should be considered for the detecting small intestine metastases.

**Table 1:** Reported cases of small intestine metastasis from malignant pleural mesothelioma

No	Author	Year	Age	Sex	Treatment of MPM	Duration after the diagnosis of MPM (months)	Location of small intestine metastasis	Symptom	Other distant metastasis	Follow-up (months)	Outcome
1	Kakugawa [2]	2007	62	M	Surgery	9	Muliple	Anemia	not described	Not described	Not described
2	Chen [3]	2008	73	M	None	0	Duodenum	Anemia	Not described	1	Dead
3	Gocho [4]	2010	52	M	Surgery	7 Days	Jejunum	Perforation	Not described	12	Dead
4	Martínez [5]	2010	57	M	Not discribed	0	Duodenum	Anemia	Not described	Not described	Not described
5	Liu [6]	2010	57	M	Surgery	10	Muliple	Intussusception	Colon, Mesenteric lymph node	1	Dead
6	Navarro [7]	2015	67	M	Surgery	13	Jejunum	Perforation	Mesenteric lymph node	Not described	Not described
7	Alkhayal [8]	2016	65	M	Not discribed	0	Jejunum	Perforation	Not described	Not described	Not described
8	Our case	2020	72	M	Chemotherapy	18	Jejunum	Intussusception	Lung	4	Alive

According to Marsicovetere *et al*. [9], intussusception accounts for only 1–5% of ileus cases. Adult cases of intussusception are rarer than pediatric cases as they account for 5–10% of all intussusceptions. In total, 90% of the symptomatic cases have been associated with identifiable causes, whereas the remaining 10% have been declared as idiopathic cases. Most intussusception in adults arises from the small intestine, and 50‑75% of lesions are benign. Malignant intraluminal causes of small intestine intussusception include primary leiomyosarcomas, adenocarcinoma, gastrointestinal stromal tumors, carcinoid tumors, neuroendocrine tumors and lymphomas [9]. Less commonly, small intestine metastasis may act as lead points of the intussusception. Although there were several reports of small intestine intussusception due to metastatic disease such as melanoma, lung cancer and renal cell cancer, MPM was extremely rare.

The main routes of metastasis to the small intestine include direct invasion, intraperitoneal seeding and hematogenous metastases. In this case, since there was no evidence of peritoneal dissemination, metastasis may have occurred through the hematogenous route. Distant hematogenous metastases usually appear during the late disease stages [10]. In half of the cases, the period from the diagnosis of MPM to the appearance of symptoms due to small intestine metastases has passed ~1 year (Table 1). Histologically, MPM is classified as epithelioid (60%), biphasic (30%) or sarcomatoid (10%). Sarcomatoid mesothelioma is associated with more frequent distant metastases than the others. Although this case was an epithelioid type MPM with a relatively good prognosis, distant metastasis might have occurred, with a long-term survival of a year and a half.

In conclusion, MPM is a disease that can cause metastasis to the small intestine. Therefore, if a patient with MPM develops abdominal pain, metastasis to the small intestine should be considered.

## CONFLICT OF INTEREST STATEMENT

None declared.

## FUNDING

None.
